# Decreased Serum Level of miR-146a as Sign of Chronic Inflammation in Type 2 Diabetic Patients

**DOI:** 10.1371/journal.pone.0115209

**Published:** 2014-12-12

**Authors:** Lucy Baldeón R., Karin Weigelt, Harm de Wit, Behiye Ozcan, Adri van Oudenaren, Fernando Sempértegui, Eric Sijbrands, Laura Grosse, Wilma Freire, Hemmo A. Drexhage, Pieter J. M. Leenen

**Affiliations:** 1 Department of Immunology, Erasmus MC, University Medical Center, Rotterdam, The Netherlands; 2 Department of Internal Medicine, Erasmus MC, University Medical Center, Rotterdam, The Netherlands; 3 Department of Immunology, Central University of Ecuador, Quito, Ecuador; 4 Institute of Research in Health and Nutrition, University San Francisco de Quito, Quito, Ecuador; 5 Department of Psychiatry, University of Münster, Münster, Germany; 6 Prometeo Program SENESCYT, Central University of Ecuador and Universidad de las Fuerzas Armadas, Quito, Ecuador; Louisiana State University Health Sciences Center, United States of America

## Abstract

**Background:**

There is increasing evidence that chronic inflammation is an important determinant in insulin resistance and in the pathogenesis of type 2 diabetes (T2D). MicroRNAs constitute a newly discovered system of cell regulation and in particular two microRNAs (miR-146a and miR-155) have been described as regulators and biomarkers of inflammation.

**Aim:**

To determine a putative association between the levels of miR-146a and miR-155 in serum of T2D patients, clinical parameters and serological indicators of inflammation.

**Methods:**

We performed quantitative Real Time PCR (qPCR) of microRNAs from serum (56 Ecuadorian T2D ambulatory patients and 40 non-diabetic controls). In addition, we evaluated T2D-related serum cytokines.chemokines and growth factors using a commercially available multi-analyte cytometric bead array system. We correlated outcomes to clinical parameters, including BMI, HbA1c and lipid state.

**Results:**

The Ecuadorian non-diabetic controls appeared as overweight (BMI>25: patients 85%, controls 82.5%) and as dyslipidemic (hypercholesterolemia: patients 60.7%, controls 67.5%) as the patients.

- The serum levels of miR-146a were significantly reduced in T2D patients as compared to these non-diabetic, but obese/dyslipidemic control group (mean patients 0.61, mean controls set at 1; p = 0.042), those of miR-155 were normal.
- The serum levels of both microRNAs correlated to each other (r = 0.478; p<0.001) and to leptin levels. The microRNAs did not correlate to BMI, glycemia and dyslipidemia.
- From the tested cytokines, chemokines and growth factors, we found IL-8 and HGF significantly raised in T2D patients versus non-diabetic controls (p = 0.011 and 0.023 respectively).

**Conclusions:**

This study shows decreased serum anti-inflammatory miR-146a, increased pro-inflammatory IL-8 and increased HGF (a vascular/insular repair factor) as discriminating markers of failure of glucose control occurring on the background of obesity and dyslipidemia.

## Introduction

It is well accepted that obesity and type 2 diabetes can be viewed as inflammatory disorders. Early, in the 1990s Hotamisligi et al. showed that TNF-α was present in obese individuals and animals in proportional levels to insulin resistance and they proposed a pathogenic role of inflammatory molecules, such as TNF-α, in the development of insulin resistance and diabetes [1]. To support this idea it was later shown that TNF-α was indeed capable to induce insulin resistance in lean animals [1]–[3] and that various pro-inflammatory cytokines trigger intracellular pathways such as Nuclear Factor for Kappa light chain in B-cells (NF- κB), IκB kinase-β (IKKβ) and Jun kinase (JNK) which are capable to inhibit the insulin signaling pathway [4]–[8].

Macrophages in adipose tissue as well as the adipocytes themselves are the prime source of the raised pro-inflammatory cytokines and adipokines, leading to a chronic pro-inflammatory state in obese subjects. In conjunction with these cellular responses in so-called “chronically inflamed” adipose tissue, a disturbed lipid metabolism is capable of inducing such a chronic pro-inflammatory state. High levels of Ox-LDL and low levels of HDL correlate to inflammatory activation and insulin resistance through a mechanism called lipotoxicity [4], [9]–[11]. Moreover, free fatty acids enhance the secretion of TNF-α, IL-6 and PAI-1, which stimulate macrophages to secrete more inflammatory cytokines and chemokines aggravating the feed-forward loop of inflammation [2], [11], [12]. All in all, there is a vast literature on increased levels of pro-inflammatory cytokines in the metabolic syndrome (MetS) and type 2 diabetes (T2D), and excellent reviews exist on this topic [13]–[17].

MicroRNAs represent a newly discovered level of cell regulation, functioning by inhibiting protein translation, and microRNAs have been suggested to be useful biomarkers in various pathological conditions, including diabetes [18], [19]. A substantial literature indicates that two microRNAs, i.e. miR-146a and miR-155, are key regulators of (auto)-inflammatory processes [20]–[31]. Dysregulation of these microRNAs in peripheral blood mononuclear cells (PBMC) has been implicated in diabetes [20], [32]. MiR-146a and miR-155 expression levels have been found to be significantly decreased in the PBMCs of patients with T2D as compared to control subjects and expression values correlated negatively to parameters of metabolic control (Hb1Ac, glucose) and signs of inflammation (NFκB mRNA levels in PBMC, circulatory levels of pro-inflammatory cytokines). MicroRNAs are, however, also detectable in serum and there are indications that microRNAs are very stable in this milieu [33]–[36], although they might be less stable in other milieus, such as the brain [37]. Measured in serum, they can serve as biomarkers and there is a study that has determined the level of miR-146a in the serum of T2D patients as one of the microRNAs of a set of 7 microRNAs considered to act as key regulators of the expression, production, secretion or effectiveness of insulin [38]. This study found raised levels of these 7 microRNAs when evaluated in relatively small groups (n = 19 each) of newly diagnosed T2D patients as compared to pre-diabetic individuals and T2D-susceptible individuals [38].

In the current study we determined the levels of miR-146a and miR-155a in the serum of 56 Ecuadorian T2D patients and of 40 non-diabetic controls and associated the levels of these microRNAs to parameters of glucose control, dyslipidemia, obesity and the serum level of 12 T2D-related inflammatory mediators (TNFα, IL-1β, IL-6, NFG, HGF, PAI, Resistin, CCL2, Adiponectin, Leptin, IL-8, and CCL4) using a commercially available multi-analyte cytometric bead array system, especially developed for type 2 diabetes (Milliplex Map, U.S.A.).

## Patients and Methods

### Patients

A total of 56 patients positively diagnosed with type 2 diabetes, according to the criteria of The Expert Committee on the diagnosis and clasification of Diabetes Mellitus [39], were recruited in 4 medical centers of Quito-Ecuador (Eugenio Espejo Hospital, Club de Leones Sur, Fundación Oftalmológica del Valle and Fundación de la Psoriasis) from 2009 til 2012. Patients with immune disorders, serious medical illness, recent infections (last 2 weeks), obvious vascular complications, fever, pregnancy/postpartum, use of statins and LADA patients (positive GAD-65 Abs) were excluded. Forty non-diabetic controls taken from the same ethnic and societal background, not suffering from important medical disorders (including acute infection) served as controls. They were included at the same time as the patients and had to be over 30 years of age and preferably of the same gender as the patients. The Medical Ethical Review Committee of the Ecuadorian Corporation of Biotechnology Quito, Ecuador approved the study. Written informed consent was obtained from the patients and controls in the study. The Ethic Committee of the Central University also validated the ethical approval of the study. The Ecuadorian Ministry of Health (MSP) gave the respective permit to export and process the samples in Erasmus MC, Rotterdam, The Netherlands.

### Serum cytokines and lipid profile

In the morning fasting venous peripheral blood (10 mL) was collected in a clotting tube and processed within 4 hours. Serum was frozen and stored at minus 80°C for approximately 24 months before testing. The levels of TNFα, IL-1β, IL-6, NGF, HGF, PAI, Resistin, CCL2 (MCP-1), Adiponectin, Leptin, IL-8, and MIP1β (CCL4) were measured by flow cytometry (BD LSR II Biosciences, California, and EE.UU.) using a commercially available multi-analyte cytometric bead array system (Milliplex Map, U.S.A.). The data were analyzed using a 5-parameter logistic method for calculating analyte concentrations in samples (MAGPIX with xPONENT software, Luminex, Austin, USA). Undetectable serum analyte levels were considered as 0 pg/ml and included in the statistical analysis. The lipid profile was performed according to standard lab procedures in Quito-Ecuador (AMCOR laboratory) and assays were validated in Erasmus MC.

### MicroRNA quantitative real-time PCR (qPCR)

Total RNA was isolated from serum using the Qiagen miRNeasy kit (Qiagen, Hilden, Germany). In order to correct for variations in RNA isolation derived, we spiked-in a non-human (C. elegans) synthetic miRNA cel-miR-39 miRNA Mimic (MSY000010) into the sample before nucleic acid isolation. Subsequently, specific stem-looped reverse transcription primers were used to obtain cDNA for mature microRNAs. The RNA was reverse transcribed using the TaqMan MicroRNA Reverse Transcription Kit from Applied Biosystems, The Netherlands (ABI). PCR was performed using pre-designed TaqMan microRNA assays and TaqMan Universal Master Mix, NoAmpEraseUNG, with an ABI 7900 HT real-time PCR machine. The PCR conditions were 2 min at 50°C, 10 min at 95°C, followed by 40 cycles of 15 s at 95°C, and finally 1 min at 60°C. The spiked-in syn-cel-miR-39 goes through the entire RNA isolation process and serves as endogenous control for data normalization.

### TaqMan assay data processing

SDS software (ABI) was used to collect the data and the RQ Manager Program (ABI) was used to assign, check and standardize C_T_ values. Data Assist software was used to normalize the data to the syn-cel-miR-39. For threshold cycles below 40, the corresponding microRNA was considered detected, higher cycle numbers were not included in the calculations. The results were represented using the ddCT method (2 ^–ddCT^, User Bulletin, ABI).

### Data analysis

Statistical analysis was performed using SPSS 20 (IBM, Inc.) package for Windows. Data were tested for normal distribution using the Kolmogorov-Smirnov test. The Grubbs' test for outlier detection was applied (http://graphpad.com/support/faqid/1598/). Depending on the distribution pattern and the total number of subjects, parametric (normal distribution, independent t test) or nonparametric group comparison (Mann-Whitney U test) were applied. Correlations were determined by Spearman correlation. Level of significance were set at p = 0.05 (two tailed). A dendrogram visualizing associations was constructed in SPSS using hierarchical cluster analysis of the serum cytokines using the between-groups linkage method. Hierarchical regression analysis was used to test if means of miR-146a, IL-8 and HGF were significantly different between Non diabetic controls and T2D patients, when controlling for BMI and lipids. Graphs were designed with GraphPad Prism 5.04 (GraphPad Software, Inc.) for Windows.

## Results

### Patient and control characteristics

Table 1 shows the number of patients and non-diabetic controls used for this study and their ages, gender, HbA1c/hyperglycemia, BMI, lipid profile and medication. As expected, the T2D patients had a significantly higher fasting glucose and HbA1c level as the non-diabetic controls. 70% of the patients used oral anti-diabetic treatment and 30% used insulin. Of the patients 61% had a history of cardiovascular disease, while 48% had a family history (1^st^, 2^nd^ degree) of diabetes (values were 29% and 29% respectively for the non-diabetic control group).

**Table 1:** Patients and Non-diabetic controls characteristics.

	Controls		T2D		Controls
					Vs.
					T2D
					p- Value
Group size n	40		56		
Age mean (range)	54 (32–87)		62 (38–85)		**0.002**
**Gender**					
Female n (%)	28 (70%)		34 (61%)		NA
Male n (%)	12 (30%)		22 (39%)		NA
**Comorbidities**					
Cardiovascular diseases n (%)	29%		61%		NA
			
Familiar antecedents of diabetes n (%)	29%		48%		NA
			
BMI mean (range) %	29.3 (23–42)	Normal 17,5%	29.2 (22–39)	Normal 14,8%	0,86
		Overweight 47,5%		Overweight 46,4%	
		Obese 35%		Obese 38,8%	
**Glucose state**					
Fasting Glucose mg/dL	86 (60.9–180.5)	Normal 95%	144 (69–397)	Normal 51.8%	0.00**
mean (range) %					
		High 5%		High 48.2%	
HbA1C	5.7 (3.9–6.7)	Normal 95%	7.1 (4.8–12.5)	Normal 35.7%	0.00**
mean (range) %					
		High 5%		High 64.3%	
**Lipid Profile**					
Cholesterol mg/dL	235 (131–328)	Normal 32.5%	233 (143–436)	Normal 39.3%	0,92
mean (range) %					
		High 67.5%		High 60.7%	
TG mean mg/dL	200 (92–547)	Normal 62.5%	197 (76–411)	Normal 66.1%	0,88
mean (range) %					
		High 37.5%		High 33.9%	
HDL mean mg/dL	41.5 (25–65)	Normal 45%	43 (18–70)	Normal 58.9%	0,71
					
mean (range) %		Low 55%		Low 41.1%	
LDL mg/dL	155 (78–266)	Normal 57.5%	153 (77–361)	Normal 62.5%	0,92
mean (range) %					
		High 42.5%		High 37.5%	
**Medication**					
Oral Anti-diabetic treatment	0%		70%		
			
Insulin treatment	0%		30%		
			
Aspirin	21%		30%		
			
Statins (%)	0%		0%		
			

Values in bold denote a significant difference between two groups.

Table 1 shows the number of patients and controls used in this study and their ages, gender, comorbidities, HbA1c/hyperglycemia, BMI, lipid profile and medication use.

With regard to the non-diabetic control group, we selected the controls by asking hospital staff (60%) and accompanying care takers (40%) to volunteer to donate blood at the same time as the patients were investigated. Controls needed to be over 30 years of age, while we tried to match as much as possible for gender. Table 1 show that we did not completely succeed in matching for age, since our controls were on average 8 years younger than the T2D patients. Gender distribution was not significantly different with a slight over representation of women in the control group.

We found the collected non-diabetic controls to be as overweight as the patients with a normal BMI in only 17.5% of the 40 non-diabetic controls as compared to 14.8% of the 56 T2D patients. There were no differences in BMI between non-diabetic hospital staff and non-diabetic care-takers. The T2D patients and non-diabetic controls also appeared to have the same disturbed lipid profile; the non-diabetic controls had hypercholesterolemia in 67.5% of individuals, the T2D patients had a hypercholesterolemia in 60.7% of cases (for further details see Table 1). There were again no differences between non-diabetic hospital staff and non-diabetic care-takers with regard to dislipidemia.

### MicroRNA's, cytokines, chemokines and growth factors in serum

**Figure 1:**
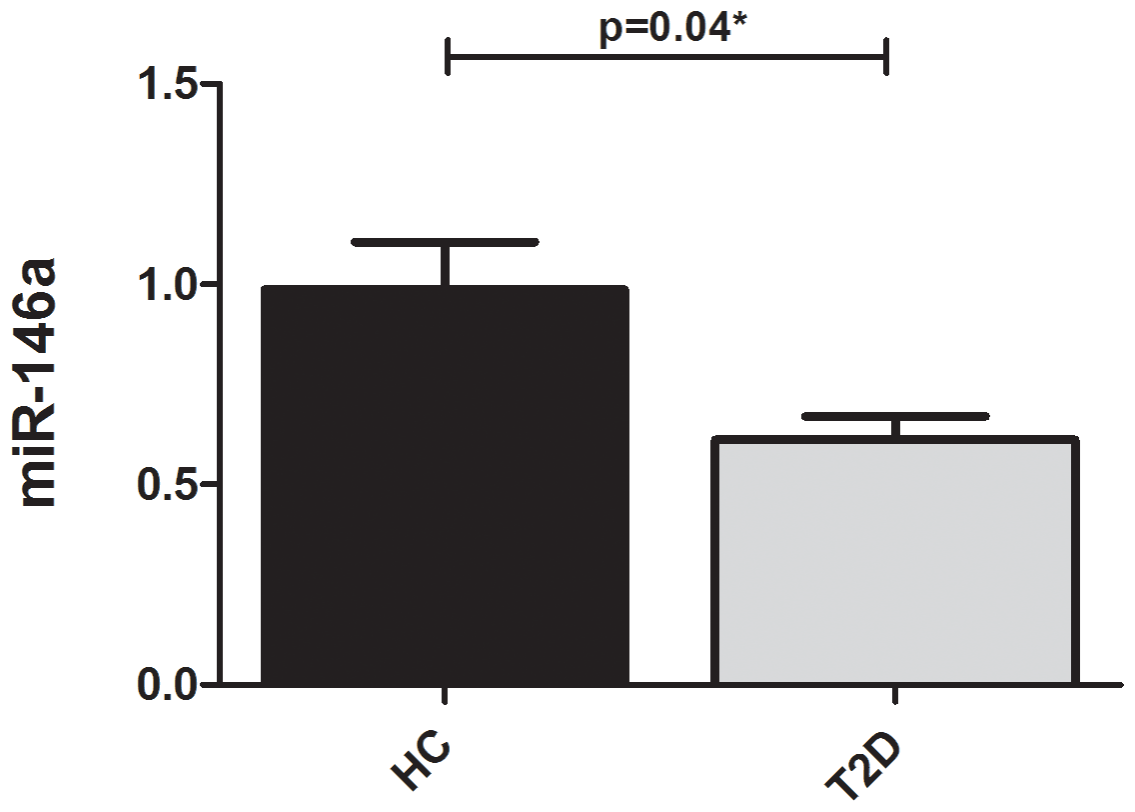
Expression level of serum miR-146a in Ecuadorian Non-diabetic controls and T2D patients. Fig. 1 shows mean and standard deviation of the fold change values of miR-146a (reference microRNA sync-cel-mir-39) in the serum of the T2D patients as compared to Non-diabetic controls. Differences between groups were tested using independent T test. Levels of significance were set at p = 0.05 (two-tailed).

Table 2 gives the mean and standard error of the mean (SEM) of the fold change values of the two tested microRNAs (146a and 155 versus the reference syn-cel-miR-39) in the serum of the T2D patients as compared to the non-diabetic controls. The serum levels of miR-146a were significantly reduced in T2D patients as compared to the non-diabetic controls (Fig. 1), those of miR-155 were not. Nevertheless there existed a good correlation between the serum levels of both microRNAs (r = 0.478; p<0.001).

**Table 2:** Cytokines, chemokines and growth factors in Non-diabetic controls and T2D patients.

				Controls vs. T2D
	T2D			*T test*
	N	Mean	(SEM)	p-Value
NGF	56	1,24	0,36	0,707
IL1beta	56	0,79	0,18	0,686
IL6	56	1,38	0,21	0,131
CCL4	56	0,92	0,1	0,775
**HGF**	56	1,34	0,09	0.023*
TNFalpha	56	1,11	0,07	0,22
Resistin	56	1,19	0,08	0,097
**IL8**	56	2,19	0,36	0.011*
Adiponectin	56	1,25	0,14	0,222
CCL2	56	1,00	0,07	0,883
**miR146a**	56	0,61	0,05	0.042*
miR155	56	0,93	0,07	0,844
Leptin	56	0,86	0,11	0,338
PAI1	56	1,17	0,23	0,565

Values in bold denote a significant difference between two groups

Group size, mean and SEM in the order of the cluster analysis. To avoid inter-assay variation, serum levels (pg/ml) were expressed in fold change compared to non-diabetic controls, the average of the Controls in each assay was set to one. Differences between groups were tested using independent T test. Levels of significance were set at p = 0.05 (two-tailed).

**Figure 2:**
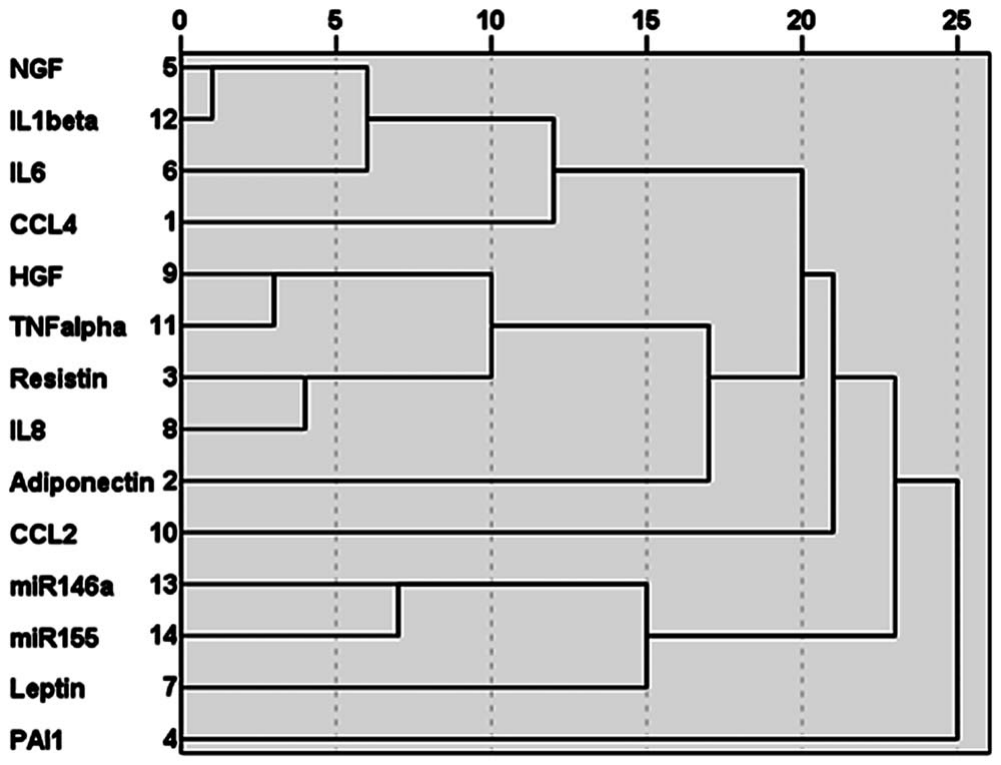
Dendrogram of unsupervised hierarchical cluster analysis of the tested serum levels of microRNAs, cytokines, chemokines and growth factors in T2D patients and Non-diabetic controls. The dendrogram shows the clustering of miR-146a and miR-155, and of the pro-inflammatory cytokines CCL4, IL-6, IL-1β and NGF and of TNF-α, IL-8, HGF and resistin.

Fig. 2 shows the unsupervised cluster analysis of the levels of the microRNAs and the tested T2D-related cytokines, chemokines and growth factors in the serum of patients and non-diabetic controls. As can be seen from the diagram there was the expected strong clustering of both microRNAs, which also clustered to some extent with leptin. With regard to the other cytokines and chemokines, there existed a clustering of the pro-inflammatory mediators CCL4, IL-6, IL-1β and NGF, and between TNF-α, IL-8, HGF and resistin. To avoid inter-assay variation, serum levels were expressed in fold changes compared to controls for each mediator.

Table 2 gives the relative levels of the tested cytokines, chemokines and other mediators in the serum of the T2D patients and non-diabetic controls in the order of the cluster analysis. From the factors determined, only the levels of IL-8 and HGF appeared to be significantly different between T2D patients and the non-diabetic controls. Both IL-8 and HGF levels were higher in the serum of the T2D patients as compared to the non-diabetic controls. Resistin was also higher in the serum of the patients, but only approached the level of significance (p = 0.09). All in all, the picture emerges of particularly the cluster of HGF, TNF-α, Resistin and IL-8 to be raised in the serum of the diabetic patients versus the non-diabetic controls.

### The correlations of the level of the microRNAs with the cytokines/chemokines/growth factors and clinical variables

We performed correlation analyses between the different parameters measured and only took correlations with a level of p<0.01 into consideration.

Since our patients and non-diabetic controls differed 8 years in age we took special notice of correlations with age. The microRNAs did not correlate with age. Of the cytokines HGF, resistin and adiponectin correlated positively to age. It is important to note that correction for age did not change the association of HGF with disease (r = 0.258, p = 0.008). Of the clinical variables HbA1c levels correlated to age.

It is also of note that the levels of miR-146a and miR-155 correlated to each other, corroborating our findings in the cluster diagram. With regard to correlations of microRNAs with cytokines we found miR-146a to correlate significantly and positively to the serum PAI level (r = 0.259; p = 0.01). There were no correlations of miR-146a and clinical variables. The serum miR-155 level correlated significant to the serum leptin level (r = 0.326, p = 0.001) and IL-8 (r = 0.268, p = 0.008).

Serum IL-8 levels correlated to HbA1c levels (r = 0.301; p = 0.003) and also positively to TNFα levels (r = 0.288, p = 0.004), which in turn correlated to HGF levels (r = 0.367; p = 0.000), corroborating our findings in the cluster diagram (Fig. 2). Positive correlations were also found between HGF and resistin levels and resistin and IL-6 levels, again corroborating the findings in the cluster diagram (Fig. 2).

Expected significant correlations were between leptin and BMI and leptin and leptin and gender.

## Discussion

In this study we determined two inflammation-related microRNAs in the serum of Ecuadorian T2D patients. We observed a significantly reduced level of one of these microRNAs, i.e. of miR-146a, in the serum of T2D patients as compared to a non-diabetic control group. Reduced expression of miR-146a is classically considered a sign of a pro-inflammatory state. Boldin et al. described that miR-146a-null mice systematically overproduce pro-inflammatory cytokines (such as TNF-α, IL-6 and IL-1β) in response to injection with a sub-lethal LPS dose. Tissue macrophages were the primary source of this enhanced pro-inflammatory cytokine production. This implicates miR-146a in attenuating macrophage inflammatory responses [40]. In agreement with these results, in vitro studies show that induction of miR-146a expression in monocyte/macrophage cell lines negatively regulates the inflammatory response [23], [41], while transfection with miR-146a inhibitors in both resting and LPS-stimulated macrophage-like cell lines had an opposite effect and resulted in an up-regulation of these inflammation-related genes. Collectively these data show that miR-146a is a strong down regulator of the production of classical inflammatory compounds in macrophages.

We also found the level of serum IL-8 significantly up regulated in the T2D patients as compared to the non-diabetic controls in agreement with previous findings of Herder et al [42]. IL-8 is considered a primary cytokine for M1 inflammatory macrophages. On the basis of these significant alterations in miR-146a and IL-8 levels we like to conclude that our study supports the concept of an activation of the inflammatory response system in T2D patients. The correlation of the IL-8 level with Hb1Ac supports the idea that chronic hyperglycemia plays at least a partial role in this activation.

A limitation of our study is that our non-diabetic control group was not matched for age to our diabetic patient group, and non-diabetic controls were on average 8 years younger than our patients; patients and non-diabetic controls did have similar readings for lipid profiles and BMI. In correlation analysis miR-146a levels and IL-8 levels appeared not to be dependent of age. When we performed hierarchical regression analysis for BMI and lipid profiles, it appeared that the disease state always was the determinant for abnormal miR-146a and IL-8 levels and that BMI and lipid profiles did virtually not determine these levels, except for IL-8 which was also determined by the cholesterol levels (see S1 and S2 Table). We are thus confident that indeed abnormal levels of miR-146a and IL-8 are determined by the T2D state in this study.

A reduced level of miR-155 has been described in the circulating leukocytes of T2D patients [32]. However we were not able to find a significant change of miR-155 in the serum of T2D patients as compared to our non-diabetic control group. We however did find a significant positive correlation between the serum levels of miR-155 and miR-146a and we found a clustered expression of both miR-146a and miR-155 with leptin in cluster analysis. Since leptin is primarily derived from adipose tissue, this might suggest that a significant proportion of the circulating microRNAs miR-146a and miR-155 is produced by activated macrophages and adipocytes in adipose tissue.

Our T2D cases lacked a significant over-expression of several classical pro-inflammatory compounds in serum: similar levels of TNF-α, IL-1β and IL-6 were found in the serum of patients and non-diabetic controls. This contrasts to previous findings by others, such as Costantini et al., who observed increased levels of IL-1α, leptin, resistin and PAI-1 in T2D patients [43]. Our negative findings might be due to the fact that our non-diabetic controls appeared to have many signs of the metabolic syndrome: BMI values were over 25 in 82.5% (average BMI 29.3), while hypercholesterolemia was present in 67.5% with raised LDL in 42.5% of non-diabetic controls. These values were similar to the ones found in the T2D cases. The Ecuadorian non-diabetic control group was composed of care-takers (40%, friends and family), and hospital staff (60%) from the Quito area. Considering this excessively high prevalence of obesity and dyslipidemia in the Quito non-diabetic control group, it is important to note that a recent healthcare report of the Ecuadorian government corroborates this high prevalence of obesity and dyslipidemia in urban Ecuadorian populations [44].

In a parallel study we have collected Dutch T2D patients and Dutch non-diabetic controls that were tested at the same time with the same multi-analyte system for cytokines and growth factors. The Dutch healthy controls had on average a BMI of 23.8 and had normal lipid values (hypercholesterolemia none, raised LDL 14%). Interestingly our Ecuadorian “healthy” control group indeed had higher levels of CCL4 and IL-6 (see S4 Table), suggesting that in particular obesity and dyslipidemia determine a higher level of these classical pro-inflammatory cytokines in serum, and not (only) the diabetes state and/or pathology per se.

Reduced levels of miR-146a have previously been found in T2D patients, be it in circulating leukocytes [20]. However, our report contrasts with another report that showed elevated levels of miR-146a in the serum of newly diagnosed T2D patients [38]. These elevated levels were found in comparison to the serum levels of pre-diabetic individuals with a disturbed OGT and T2D-susceptible individuals (family), who only had a moderate overweight (average BMI of 26) and moderate hypercholesterolemia (average 5.6 mmol/l), as had the newly T2D cases in that study. We therefore assume that the distinct status of the control population with regard to obesity and/or severe dyslipidemia might have played a role in the differences. In addition, our T2D patients had longstanding diabetes, and the stage of disease may have played a role as well.

Apart from the involvement of inflammatory miR-146a and IL-8, our study suggests an involvement of other molecular systems associated with the failure to control glucose homeostasis on the background of an already existing obesity and dyslipidemia.

First, significantly higher serum levels of HGF were found in the T2D patients as compared to the non-diabetic controls. HGF levels correlated to age, but correction for age left the association with T2D intact (similar results were obtained with corrections for BMI and dyslipidemia, see S3 Table). HGF was first described as a hepatocyte factor involved in liver regeneration after partial hepatectomy [45]. Recent evidence shows that the factor is also produced by monocytes and macrophages and that it is involved in various regeneration processes, including vascular repair and β cell growth [46]–[48]. HGF can thus be viewed as a key factor in insulin resistance-associated compensatory mechanisms at the level of the pancreatic islet by stimulating its regeneration and at the level of the vasculature by stimulating repair of hyperglycemia-damaged vessels by inducing proliferation of endothelial cells. In marked contrast, however, HGF has also been implicated with a pathogenic role in macrovascular disease as HGF levels in type 2 diabetes patients correlated positively with carotid intimal-media thickness and plaque score [49].

In addition to a higher level of HGF there was also an over-expression (non-significant, p = 0.09) of resistin in the serum of the Ecuadorian T2D cases as compared to the non-diabetic controls. Resistin was initially identified in adipocytes, but significant levels of resistin expression in humans are mainly found in immune cells, particularly monocytes [50], [51]. Resistin was first described as a factor contributing to the development of insulin resistance and diabetes in humans, but debate is still ongoing regarding its role in obesity, insulin sensitivity and the development of T2D. In addition also evidence for a pathogenic role of resistin in atherogenic vascular diseases is growing [52], [53].

In conclusion this study shows signs of chronic inflammation (decreased serum anti-inflammatory miR-146a/increased IL-8) and signs of islet and vascular repair (increased HGF) in patients with a failure to control glucose homeostasis when compared to non-diabetic controls with a similar prevalence of obesity and dyslipidemia. Our study also suggests that miR-146a can be considered as a serum biomarker of the inflammatory process linked to the failure of glucose control of the T2D state against a background of obesity and dyslipidemia.

## Supporting Information

S1 Table **Hierarchical Regression Model of miRNA-146a.** Hierarchical regression analysis for BMI and lipid profiles shows that the disease state was the determinant for abnormal miR-146a.
(DOCX)

S2 Table **Hierarchical Regression Model of IL-8.** Hierarchical regression analysis for BMI and lipid profiles shows that the disease state and cholesterol levels were the determinant for abnormal IL-8.
(DOCX)

S3 Table **Hierarchical Regression Model of HGF.** Hierarchical regression analysis for BMI and lipid profiles shows that the disease state was the determinant for abnormal HGF.
(DOCX)

S4 Table **Cytokines and chemokines mediators of Ecuadorian Non-diabetic controls and Dutch healthy controls.** Group size, median, inter-quartile range (IQR) and p-values obtained by Mann—Whitney U-test is represented. Serum levels (pg/ml) are shown in the order of the cluster analysis. In a parallel study we have collected Dutch healthy controls that were tested at the same time with the same multi-analyte system for cytokines and growth factors. Ecuadorian non-diabetic controls showed higher levels of the classical pro-inflammatory cytokines (CCL4 and IL-6).
(DOCX)

S1 Data **Raw data points of the tested serum levels of microRNAs, cytokines, chemokines and growth factors of T2D patients and Non-diabetic controls.** The levels of TNFα, IL-1β, IL-6, NGF, HGF, PAI, Resistin, CCL2 (MCP-1), Adiponectin, Leptin, IL-8, and MIP1β (CCL4) were measured by flow cytometry (BD LSR II Biosciences, California, and EE.UU.) using a commercially available multi-analyte cytometric bead array system (Milliplex Map, U.S.A.). MicroRNA quantitative real-time PCR (qPCR) was performed using pre-designed TaqMan microRNA, with an ABI 7900 HT real-time PCR machine. SDS software (ABI) was used to collect the data.
(ZIP)
