# Supplementary material for: Decreased Serum Level of miR-146a as Sign of Chronic Inflammation in Type 2 Diabetic Patients
Source: PLoS One. 2014 Dec 12;9(12):e115209. doi: 10.1371/journal.pone.0115209 (PMC4264887; doi:10.1371/journal.pone.0115209)
Supplement: S1 Table — Hierarchical Regression Model of miRNA-146a. Hierarchical regression analysis for BMI and lipid profiles shows that the disease state was the determinant for abnormal miR-146a. (DOCX) [file pone.0115209.s001.docx]

**Table S1**.Hierarchical Regression Model of miRNA-146a

|  | **R** | **R^2^** | **R^2^** | **B** | **SE** | **β** | **t** |
| --- | --- | --- | --- | --- | --- | --- | --- |
|  |  |  | **Change** |  |  |  |  |
| ***Model 1*** | 0.213 | .045 | .000 |  |  |  |  |
| Disease |  |  |  | -.245 | .118 | -.212***** | -2.070 |
| BMI |  |  |  | -.003 | .015 | -.022 | -.217 |
| ***Model 2*** | 0.211 | .044 | .001 |  |  |  |  |
| Disease |  |  |  | -.239 | .117 | -.208***** | -2.051 |
| Cholesterol |  |  |  | .000 | .001 | .031 | .302 |
| ***Model 3*** | 0.214 | .046 | .003 |  |  |  |  |
| Disease |  |  |  | -.237 | .117 | -.206***** | -2.032 |
| HDL |  |  |  | -.003 | .005 | -.050 | -.495 |
| ***Model 4*** | 0.21 | .044 | .001 |  |  |  |  |
| Disease |  |  |  | -.239 | .117 | -.208***** | -2.050 |
| LDL |  |  |  | .000 | .001 | .029 | .285 |
| ***Model 5*** | 0.211 | .044 | .001 |  |  |  |  |
| Disease |  |  |  | -.240 | .117 | -.209***** | -2.061 |
| Tryglicerids |  |  |  | .000 | .001 | -.032 | -.317 |

**Note.** Statistical significance: *p< .05;**p< .01; ***p< .001

**Table S1.** *Hierarchical Regression Model of miRNA-146a.* Hierarchical regression analysis for BMI and lipid profiles shows that the disease state was the determinant for abnormal miR-146a.
