# Supplementary material for: Decreased Serum Level of miR-146a as Sign of Chronic Inflammation in Type 2 Diabetic Patients
Source: PLoS One. 2014 Dec 12;9(12):e115209. doi: 10.1371/journal.pone.0115209 (PMC4264887; doi:10.1371/journal.pone.0115209)
Supplement: S2 Table — Hierarchical Regression Model of IL-8. Hierarchical regression analysis for BMI and lipid profiles shows that the disease state and cholesterol levels were the determinant for abnormal IL-8. (DOCX) [file pone.0115209.s002.docx]

| **Table S2.** *Hierarchical Regression Model of IL-8* | | | | | |  |  |
| --- | --- | --- | --- | --- | --- | --- | --- |
|  | **R** | **R^2^** | **R^2^** | **B** | **SE** | **β** | **t** |
|  |  |  | **Change** |  |  |  |  |
| ***Model 1*** | 0.272 | .074 | .000 |  |  |  |  |
| Disease |  |  |  | 1.207 | .448 | .272****** | 2.694 |
| BMI |  |  |  | .008 | .056 | .014 | .143 |
| ***Model 2*** | 0.324 | .105 | .038 |  |  |  |  |
| Disease |  |  |  | 1.133 | .434 | .256****** | 2.614 |
| Cholesterol |  |  |  | -.008 | .004 | -.195***** | -1.983 |
| ***Model 3*** | 0.279 | .078 | .011 |  |  |  |  |
| Disease |  |  |  | 1.168 | .440 | .264****** | 2.652 |
| HDL |  |  |  | -.021 | .020 | -.104 | -1.043 |
| ***Model 4*** | 0.299 | .089 | .022 |  |  |  |  |
| Disease |  |  |  | 1.135 | .437 | .257****** | 2.595 |
| LDL |  |  |  | -.006 | .004 | -.148 | -1.498 |
| ***Model 5*** | 0.287 | .083 | .015 |  |  |  |  |
| Disease |  |  |  | 1.138 | .439 | .258****** | 2.592 |
| Tryglicerds |  |  |  | -.003 | .003 | -.123 | -1.242 |

**Note.** Statistical significance: *p< .05;**p< .01; ***p< .001

**Table S2.** *Hierarchical Regression Model of IL-8.* Hierarchical regression analysis for BMI and lipid profiles shows that the disease state and cholesterol levels were the determinant for abnormal IL-8.
