# Supplementary material for: Decreased Serum Level of miR-146a as Sign of Chronic Inflammation in Type 2 Diabetic Patients
Source: PLoS One. 2014 Dec 12;9(12):e115209. doi: 10.1371/journal.pone.0115209 (PMC4264887; doi:10.1371/journal.pone.0115209)
Supplement: S3 Table — Hierarchical Regression Model of HGF. Hierarchical regression analysis for BMI and lipid profiles shows that the disease state was the determinant for abnormal HGF. (DOCX) [file pone.0115209.s003.docx]

| **Table S3.** *Hierarchical Regression Model of HGF* | | | | | |  |  |
| --- | --- | --- | --- | --- | --- | --- | --- |
|  | **R** | **R^2^** | **R^2^** | **B** | **SE** | **β** | **t** |
|  |  |  | **Change** |  |  |  |  |
| ***Model 1*** | 0.244 | .060 | .003 |  |  |  |  |
| Disease |  |  |  | .351 | .151 | .236***** | 2.320 |
| BMI |  |  |  | -.011 | .019 | -.057 | -.564 |
| ***Model 2*** | 0.232 | .054 | .000 |  |  |  |  |
| Disease |  |  |  | .342 | .149 | .231***** | 2.292 |
| Cholesterol |  |  |  | .000 | .001 | -.009 | -.089 |
| ***Model 3*** | 0.232 | .054 | .000 |  |  |  |  |
| Disease |  |  |  | .341 | .149 | .230***** | 2.283 |
| HDL |  |  |  | .001 | .007 | .020 | .199 |
| ***Model 4*** | 0.231 | .054 | .000 |  |  |  |  |
| Disease |  |  |  | .342 | .149 | .231***** | 2.294 |
| LDL |  |  |  | 1.704E-06 | .001 | .000 | .001 |
| ***Model 5*** | 0.259 | .067 | .013 |  |  |  |  |
| Disease |  |  |  | .339 | .148 | .230***** | 2.292 |
| Tryglicerids |  |  |  | -.001 | .001 | -.116 | -1.160 |

**Note.** Statistical significance: *p< .05;**p< .01; ***p< .001

**Table S3.** *Hierarchical Regression Model of HGF.* Hierarchical regression analysis for BMI and lipid profiles shows that the disease state was the determinant for abnormal HGF.
