# Supplementary material for: Decreased Serum Level of miR-146a as Sign of Chronic Inflammation in Type 2 Diabetic Patients
Source: PLoS One. 2014 Dec 12;9(12):e115209. doi: 10.1371/journal.pone.0115209 (PMC4264887; doi:10.1371/journal.pone.0115209)
Supplement: S4 Table — Cytokines and chemokines mediators of Ecuadorian Non-diabetic controls and Dutch healthy controls. Group size, median, inter-quartile range (IQR) and p-values obtained by Mann—Whitney U-test is represented. Serum levels (pg/ml) are shown in the order of the cluster analysis. In a parallel study we have collected Dutch healthy controls that were tested at the same time with the same multi-analyte system for cytokines and growth factors. Ecuadorian non-diabetic controls showed higher levels of the classical pro-inflammatory cytokines (CCL4 and IL-6). (DOCX) [file pone.0115209.s004.docx]

**Table S4.** *Cytokines, chemokines and growth factors of Ecuadorian non-diabetic controls and Dutch healthy controls.*

| **Controls Ecuador** | | | | **Controls Netherlands** | | | |
| --- | --- | --- | --- | --- | --- | --- | --- |
|  |  |  |  |  |  |  | **(Ecu Cont**  **vs.**  **Dut Cont)** |
|  |  |  |  |  |  |  | ***Mann Whitney T*** |
|  | **N** | **Median** | **(IQR)** | **N** | **Median** | **(IQR)** | **p-Value** |
| **NGF** | 24 | 1.00 | 1.00 | 15 | 4.46 | 2.07 | **0.000**** |
| **IL-1beta** | 12 | 1.00 | 0.75 | 15 | 1.07 | 0.89 | 0.755 |
| **IL-6** | 34 | 4.50 | 3.00 | 15 | 0.98 | 1.73 | **0.000**** |
| **CCL4** | 34 | 95 | 101 | 15 | 52 | 77 | *0.065* |
|  |  |  |  |  |  |  |  |
| **HGF** | 34 | 733 | 414 | 15 | 829 | 693 | 0.374 |
| **TNFalpha** | 33 | 4.00 | 2.00 | 15 | 4.36 | 3.23 | 0.553 |
| **Resistin** | 34 | 36025 | 16895 | 15 | 53602 | 24980 | **0.002*** |
| **IL-8** | 34 | 6.00 | 3.25 | 15 | 5.90 | 2.48 | 0.420 |
|  |  |  |  |  |  |  |  |
| **Adiponectin** | 34 | 2325 | 1902 | 15 | 2855 | 1723 | 0.288 |
| **CCL2** | 34 | 304 | 177 | 15 | 232 | 149 | *0.083* |
|  |  |  |  |  |  |  |  |
| **Leptin** | 34 | 8599 | 8458 | 15 | 8834 | 10939 | 0.879 |
| **PAI-1** | 34 | 88898 | 31182 | 15 | 89170 | 39978 | 0.544 |

*Values in bold denote a significant difference between two groups.*

**Table S4.** *Cytokines and chemokines mediators of Ecuadorian Non-diabetic controls and Dutch healthy controls.* Group size, median, inter-quartile range (IQR) and p-values obtained by Mann—Whitney U-test is represented. Serum levels (pg/ml) are shown in the order of the cluster analysis. In a parallel study we have collected Dutch healthy controls that were tested at the same time with the same multi-analyte system for cytokines and growth factors. Ecuadorian non-diabetic controls showed higher levels of the classical pro-inflammatory cytokines (CCL4 and IL-6).
